# Evaluation of Three Swabbing Devices for Detection of *Listeria monocytogenes* on Different Types of Food Contact Surfaces

**DOI:** 10.3390/ijerph110100804

**Published:** 2014-01-08

**Authors:** Evy Lahou, Mieke Uyttendaele

**Affiliations:** Laboratory of Food Microbiology and Food Preservation, Department of Food Safety and Food Quality, Ghent University, Coupure Links 653, Ghent 9000, Belgium; E-Mail: ieke.uyttendaele@ugent.be

**Keywords:** environmental sampling, swabbing devices, *Listeria monocytogenes*, food processing environment

## Abstract

*Listeria monocytogenes* can adhere to different types of food contact surfaces within a food processing environment. Therefore, environmental sampling devices should be capable of detecting unacceptable contamination. In this study, a sponge-stick, foam spatula and an environmental swab were evaluated on their ability to detect low concentrations of *L. monocytogenes* on different types of food contact surfaces. A cocktail of four *L. monocytogenes* serotypes was inoculated with a concentration of 100 CFU/250 cm^2^ onto stainless steel (SS), high density polyethylene (HDPE) and rubber surfaces in a 250 cm^2^ area. Immediately after inoculation and after 1 h exposure, the surfaces were swabbed with the different swabbing devices. The results of the study show only minor differences in the ability of the swabbing devices to detect *L. monocytogenes*. All devices were capable to detect the contamination immediately after inoculation. However, when the surfaces were allowed to air-dry for 1 h, *L. monocytogenes* was undetected in 11.1% of the samples (*n* = 27) with the sponge stick, in 7.4% of the samples (*n* = 27) with the foam spatula and in 3.7% of the samples (*n* = 27) with the environmental swab, especially on SS surfaces. The detection ability of the different devices for *L. monocytogenes* can be concluded to be rather high on different types of food contact surfaces.

## 1. Introduction

A number of studies have been devoted to show the ability of *Listeria monocytogenes* to attach to food contact surfaces consisting of stainless steel, high-density polyethylene (HDPE) or rubber [1,2,3,4]. This dangerous foodborne pathogen is the causative agent of listeriosis, a severe disease with hospitalization and mortality rates ranging from 16% to 21% [5,6,7,8,9]. Human infection usually occurs via contaminated raw and processed ready-to-eat (RTE) foods such as cheese, smoked fish, meat products and deli-salads [9,10,11]. However, the ability of this bacterium to survive and grow under anaerobic conditions as well refrigerator temperatures, and its high tolerance to salt, allows it to thrive in food processing environments [11,12]. Especially in meat, dairy and seafood processing plants is *L. monocytogenes* commonly isolated from the food processing environment where it can exist in biofilms [9,10,13,14,15]. These biofilms can occur on food handling or food processing surfaces (e.g., cutting boards, slicing blades, conveyer belts and stainless steel equipment) or in food processing or storage areas (e.g., drains, ventilation, floors, refrigerators) [9,13,16]. The presence of biofilms in food manufacturing and processing facilities can lead to contamination of food products placed on these contaminated surfaces or handled in contaminated areas [9,17]. Moreover, it was suggested that *L. monocytogenes* strains sequestered within biofilms associated with uneven food contact surfaces may be the most important factor for product contamination [14]. The environment in which foods are prepared or handled can thus have a significant impact on the microbiological safety of food products.

To prevent unacceptable contamination of RTE food products, environmental sampling on a routine basis is recommended in the frame of the EC Regulation 2073/2005 defining microbiological criteria for foods [18]. Such environmental sampling aims at detection and elimination of persistent strains and is mostly performed with contact plates, traditional swabs and pre-moistened sponges or wipes according to ISO 18593 [19,20]. However, this ISO document does not give guidance specific to *L. monocytogenes* detection. Moreover, an ineffective sampling technique or device may result in the non-detection of *L. monocytogenes* when it is present [21]. This will prevent the implementation of corrective actions and will give a false sense of security. Recently, several studies have been performed to evaluate different sampling procedures for *L. monocytogenes* [12,19,22,23,24]. These procedures involved mini-rollers, swabs, sponges, tissues, petrifilm and RODAC plates. From these procedures, swabbing is a widely used sampling method for detection of *L. monocytogenes* [25,26]. However, studies which compare the efficacy of several environmental swab or sponge types to detect low numbers of *L. monocytogenes* on different types of food contact surfaces, are rare. Therefore, the objective of this study is to compare several environmental swabbing devices (foam spatula, environmental swab and sponge stick) in their ability to detect low concentrations of *L. monocytogenes* on different types of food contact surfaces.

## 2. Experimental Section

### 2.1. Preparation of Test Surfaces

Three types of surfaces common to the food processing environment were used as model surfaces for testing. Scratch-free stainless steel (SS, grade 316), neoprene rubber and high density polyethylene (HDPE, type 300) plates were ordered from Baudoin (Mol, Belgium) and cut into coupons of 16 × 16 cm. Prior to inoculation, the surfaces were cleaned and disinfected. The stainless steel plates were autoclaved at 121 °C for 15 min. The rubber and HDPE plates were rinsed with boiling water for 5 min and disinfected with 70% ethanol (Merck, Darmstadt, Germany) to accomplish sterile starting conditions.

### 2.2. Preparation of Inoculum

Four strains of *L. monocytogenes* were used to contaminate the test surfaces: *L. monocytogenes* serotypes 1/2a (LFMFP 511, clinical isolate), 1/2b (LFMFP 417, food isolate), 1 (LFMFP 482, food isolate) and 4b (LFMFP 423, food isolate). These strains were taken from the reference stock stored at −80 °C and were cultured in Tryptone Soy Broth (TSB, Oxoid, Basingstoke, UK), supplemented with 0.6% yeast extract (YE, Oxoid) for 24 h at 37 °C. A stock culture was kept at 4 °C on TSA supplemented with 0.6% YE. Working cultures of *L. monocytogenes* were prepared by loop inoculation of each serotype in 10 mL of fresh TSBYE and incubation for 24 ± 2 h at 37 °C. After this period, 100 µL of each culture was transferred to 10 mL of fresh TSBYE and incubated at 37 °C for 24 ± 2 h. These culture conditions were found to yield approximately 10^8^–10^9^ cfu·mL^−1^. Each serotype was pooled into a *L. monocytogenes* cocktail in equal concentrations to be used in this study. The cocktail was serially diluted in Buffered Peptone Water (BPW, Oxoid) for a concentration of ca 10^2^ cfu·mL^−1^. Culture purity was checked on Tryptone Soy Agar (TSA, Oxoid), supplemented with 0.6% YE. The strains were confirmed on Agar Listeria Ottavani & Agosti (ALOA; Biolife, Milan, Italy).

### 2.3. Application of the Inoculum on the Test Surfaces

The prepared inoculum was spotted in 10 drops of 100 µL on each test surface and uniformly spread with a sterile, disposable “hockey stick” shape spatula to obtain an inoculum level of ca 10^2^ *L. monocytogenes* CFU/250 cm^2^. To verify the number of cells inoculated on the surfaces, a plate count was conducted on TSAYE.

### 2.4. Sampling Devices and Sampling Procedure

The tested swabbing devices were: (1) a sponge-stick pre-moistened with Buffered Peptone Water Broth (3M^TM^ Sponge-Stick, 3M, Diegem, Belgium); (2) a pre-moistened environmental swab (3M^TM^ Enviro Swab, 3M, Diegem, Belgium) and (3) a Copan foam spatula (Novolab, Geraardsbergen, Belgium) pre-moistened with 10 mL BPW. For each surface type and each swab type, 9 inoculated coupons were either sampled immediately after inoculation, while they were still wet, or after they had been allowed to air-dry for 1 h.

#### 2.4.1. 3M^TM^ Sponge-Stick

**Figure 1:**
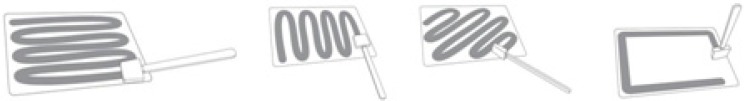
This figure shows the technique of swabbing with an overlapping “S” pattern. The tip of the swab/sponge can be used to wipe the perimeter of the sampling area, resulting in an optimal use of the swab/sponge surface.

An envelope containing a pre-moistened sponge stick was opened and the stick was aseptically removed. Swabbing was performed using an overlapping “S” pattern to cover the entire surface with horizontal strokes. Next, the swab was rotated and the same area was swabbed again using vertical “S”-strokes followed by swabbing using diagonal “S”-strokes. The tip of the device was used to wipe the perimeter of the sampling area (Figure 1). The sponge-stick was placed into the bag and the stick was bending to break off, allowing the sponge to drop in to the bag. The sponge was enriched with 225 mL of half fraser broth (Biomérieux, Brussels, Belgium) and subsequently incubated for 24 ± 2 h at 30 °C. An aliquot of 0.1 mL was spread on ALOA agar and incubated for 24 ± 2 h at 37 °C.

#### 2.4.2. Copan Foam Spatula

The foam spatula was pre-moistened with 10 mL BPW before sampling. Swabbing was performed in the same way as for the sponge-stick (Figure 1). The foam spatula was placed into a sterile sampling bag (Novolab, Geraardsbergen, Belgium) and the stick was bending to break off, allowing the foam to drop in the bag. The foam was enriched with 225 mL of half fraser broth and subsequently incubated for 24 ± 2 h at 30 °C. An aliquot of 0.1 mL was spread on ALOA agar and incubated for 24 ± 2 h at 37 °C.

#### 2.4.3. 3M^TM^ Enviro Swab

The design of the 3M^®^ Enviroswab enabled the use of a rolling motion during swabbing, which maximizes the swab contact with the surface. Swabbing was performed using an overlapping “S” pattern to cover the entire surface with horizontal, vertical and diagonal “S”-strokes. The tip of the device was used to wipe the perimeter of the sampling area. The swab was placed back in its tube, enriched with 20 mL half Fraser broth and subsequently incubated for 24 ± 2 h at 30 °C. An aliquot of 0.1 mL was spread on ALOA agar and incubated for 24 ± 2 h at 37 °C.

### 2.5. Data Analysis

All swab experiments utilized triplicate coupons and were replicated three times. Data were analyzed using SPSS Statistics (version 20, IBM, Armonk, NY, USA). The Pearson’s chi-squared test (χ^2^) was used to determine significant differences between the used swabs. A significance level of *p* = 0.05 is used.

## 3. Results and Discussion

The main purpose of environmental sampling for *L. monocytogenes* is to detect and eliminate persistent strains of this pathogen in the food processing environment. Studies of Autio *et al*. [27] and Dimitrijevíc *et al*. [14] have been demonstrating that the prevalence of *L. monocytogenes* in the environment of cold-smoked trout processing plants ranges from 6.06%–30%. Moreover, Kovačević *et al*. [28] showed a correlation between the presence of *L. monocytogenes* in RTE food products and the presence of *L. monocytogenes* in the environment of these food processing industries. Tompkin [16] also indicated recontamination as primary source of *L. monocytogenes* in many commercially prepared RTE foods. Therefore environmental monitoring is necessary to prevent product contamination and to reduce the likelihood of human infection. Moreover, end product testing becomes of little value for assessing and verifying control because contamination prevalences of food products are low [16].

Although no ideal sampling method exists, the Food Safety and Inspection Service of the USDA recommends sponges as the environmental sampling technique [29]. However, many different types are available on the market today. Moore *et al*. [30] concluded from their study that careful selection of swabbing materials can increase the sensitivity of traditional microbiological analysis. Therefore, we evaluated swabbing devices composed out of different materials on three types of surfaces common to the food processing area. Because the food processing environment is frequently contaminated with low numbers of *L. monocytogenes*, the swabbing devices were tested on their ability to detect low numbers of this pathogenic bacterium. As Gómez *et al*. [19] determined in their study that scratched surfaces do not necessarily allow a higher buildup of microorganisms than intact surfaces, it was decided to leave the test surfaces intact for the analyses.

Table 1 summarizes the results of the different swabbing devices in their ability to detect low concentrations of *L. monocytogenes* on the different food contact surfaces. In general, the sponge-stick was able to detect *L. monocytogenes* on stainless steel, HDPE and rubber compounds in respectively 83%, 100% and 100% of the samples (*n* = 18), independent of the time of sampling. The detection of *L. monocytogenes* with the foam spatula from stainless steel, HDPE and rubber compounds was possible in respectively 94%, 94% and 100% of the samples (*n* = 18). With the environmental swab, detection was possible on all tested HDPE and rubber surfaces, although detection on stainless steel was possible in 94% of the samples (*n* = 18).

### 3.1. Influence of Time of Sampling

If time of sampling is taken into account, a significant difference (*p* = 0.013) can be observed between the two sampling times. At time zero (T0), the time of swabbing immediately after inoculation, the effectiveness of each swabbing device was affirmed as shown by the positive results for each repetition. Each swabbing device could detect *L. monocytogenes* independently of the type of food contact surface. However, when the surfaces were allowed to air-dry for 1 h (T1), minor differences can be observed between the different devices and the different surfaces. As can be seen in Table 1, the sponge-stick failed three out of 27 times (11.1%) in detection of *L. monocytogenes*, while the foam spatula failed two out of 27 times (7.4%) and the environmental swab failed one out of 27 times (3.7%).

**Table 1:** Results of the different swabbing devices when used for swabbing on stainless steel (SS), high density poly-ethylene (HDPE) and rubber. T0 is the sampling time immediately after inoculation, whilst the surface was still wet. T1 is the sampling time when the surface had been allowed to air-dry for 1 h at room temperature. “+” is used when the swabbing device could detect *L. monocytogenes*. “−” is used when the swabbing device could not detect *L. monocytogenes*.

	3M^TM^ Sponge-Stick		Copan Foam Spatula		3M^TM^ Enviro Swab	
	T0	T1	T0	T1	T0	T1
SS	+	+	+	+	+	+
	+	+	+	+	+	+
	+	+	+	−	+	+
	+	+	+	+	+	+
	+	−	+	+	+	+
	+	−	+	+	+	+
	+	+	+	+	+	+
	+	−	+	+	+	−
	+	+	+	+	+	+
HDPE	+	+	+	−	+	+
	+	+	+	+	+	+
	+	+	+	+	+	+
	+	+	+	+	+	+
	+	+	+	+	+	+
	+	+	+	+	+	+
	+	+	+	+	+	+
	+	+	+	+	+	+
	+	+	+	+	+	+
Rubber	+	+	+	+	+	+
	+	+	+	+	+	+
	+	+	+	+	+	+
	+	+	+	+	+	+
	+	+	+	+	+	+
	+	+	+	+	+	+
	+	+	+	+	+	+
	+	+	+	+	+	+
	+	+	+	+	+	+

### 3.2. Influence of State of the Surface

The effect of the state of the surface (wet or dry) on the detection of the pathogen is also remarkable. It has been previously observed that wet surfaces yield a better recovery than dry surfaces, which can be due to loss of viability of the micro-organisms by drying [12,31]. Another possibility is that *L. monocytogenes* is better attached to the surface after drying. Several studies show the ability of *L. monocytogenes* to attach quickly (<20 min) to different food contact materials [32,33]. Therefore, the influence of time of sampling is associated with the state of the surface and may explain the difference between T0 and T1.

### 3.3. Influence of Surface Type

The surface type has a small significant effect (*p* = 0.026) on the ability to detect low concentrations of *L. monocytogenes*. If the different surfaces are compared, three out of 27 times (11.1%) *L. monocytogenes* could not be detected on stainless steel surfaces, as can be seen in Table 1, while on rubber compounds no detection problems could be observed. The structure of the surface material may explain these differences between the test surfaces [4,26]. Although Rodriguez *et al*. [3] could not find a significant effect of surface roughness and stainless steel finish on the attachment ability of *L. monocytogenes*. Gómez *et al*. [19] also demonstrated that surface structure did not influence the recovery of *L. monocytogenes*. However, the surface material may influence the viability of *L. monocytogenes* cells. Silva *et al*. [4] showed a decreased viability on stainless steel surfaces in comparison with polypropylene surfaces, which may explain the difference in our results between stainless steel and HDPE, but also between stainless steel and rubber.

### 3.4. Influence of Swabbing Device

In our study, no significant difference (*p* = 0.595) could be observed between the different swabbing devices. It has been previously reported that accurate detection of microbial contaminants initially relies on the ability of the swabbing device to remove micro-organisms from the surface, followed by their effective release from the swab bud [31]. These abilities are influenced by the materials from which the swabbing device is made up [12]. However, based on these findings, it can be suggested that the different swabbing devices in this study have equal detection abilities although they are composed out of different materials. The degree of pressure applied on the swabbing device influences the recovery of bacteria as well [12]. However, this is difficult to quantify but can explain why negative results are obtained even when the same test is repeated multiple times. Because of these drawbacks in recovery of *L. monocytogenes* from surfaces as well the low repeatability of recovery experiments using the traditional swabbing technique, this study focused on detection of *L. monocytogenes* instead on quantitative recovery of this bacterium. Moreover, it is recommended to test for presence or absence of *L. monocytogenes* on the surface in food processing environments [25]. If positive results are obtained for a certain sampling point, the problem can be corrected and followed up to ensure consumer protection [16].

In summary, swabbing is influenced by the time of sampling, state of the surface and surface type. As previous mentioned, *L. monocytogenes* can adhere quickly to different types of surfaces. This attachment can be influenced by the type of surface [32,33], but other extrinsic factors such as attachment period and the broth used for inoculation may influence the degree of attachment as well [34,35]. However, loss of viability due to a limited amount of nutrients is likely to happen after longer periods of drying. Therefore, it is assumed that a stronger attachment of bacteria on stainless steel is responsible for the failures in detection after the 1h air-drying in this study. During or immediately after food processing there will be food components present which in most cases favor the attachment of *L. monocytogenes*. Takahashi *et al*. [35] have been performing experiments to evaluate the influence of attached food components on the survival of *L. monocytogenes* on stainless steel. In case of food soils of minced tuna, cabbage and ground pork better survival of *L. monocytogenes* is observed at day 0 (sampled after 2 h of visual drying). Therefore, even better attachment could have been obtained when another type of broth had been used. When the cells get better attached, it will be indeed more difficult to pick-up these cells, which may influence the performance of the swabs. However, in this case study, sampling was performed in a short time frame, limiting the effect of a stronger attachment of the cells. However, it should be taken into account when sampling in the food processing environment.

The control of *L. monocytogenes* in the food processing environment does not only rely on the appropriate sampling device, but also on an appropriate sampling plan. The French agency for food, environmental and occupational healt safety (Anses) and the European Union Reference Laboratory for *Listeria monocytogenes* (EURL Lm) have recently published some guidelines on sampling the food processing area and equipment for the detection of *L. monocytogenes* [25]. They advise that the total sampled area during a sampling campaign should be as large as possible to increase the probability to detect *L. monocytogenes*. Therefore, they recommend that the area to be sampled is at least 1,000 cm^2^ (e.g., 50 × 20 cm). In this study the sampling devices were tested on 250 cm^2^, which is smaller than the recommended sampling area size. However, the surface area size in this study is at least twice as big than the surfaces used in many other studies [19,22,23,24,34]. Due to the limited size of most swab devices, sponge sticks and environmental swabs may be well suited for sampling areas that are not easily accessible and which are at least 100 cm^2^, the minimum area recommended in ISO 18593 [20]. When bigger sampling areas (>1,000 cm^2^), as preferred, are intended to be taken up in the environmental sampling plan, it is recommended to use sponges or cloths [23]. However, swabs with head diameters of 0.5 cm such as cotton swabs, rayon swabs and alginate swabs are not suitable to swab the recommended sampling areas and should not be used for these purposes in environmental sampling plans, even if it is to sample hard to reach areas. It is also recommended to sample the environment during processing (with a least 2 h of production) of foods or at the end of the production run (but before cleaning and disinfection), because cells remaining in harbourage sites (biofilms) will be more accessible to sampling once dislodged during processing because equipment vibrates and/or because foods and liquids come in contact with harbourage sites. Thus increasing the probability of detecting a persistent strain [25].

## 4. Conclusions

The different swabbing devices used in this study are able to detect low concentrations of *L. monocytogenes* on stainless steel, high-density polyethylene and rubber. Remarkable differences were observed between the two sampling times due to the state of the surface. Only minor differences could be detected between the different surfaces, which can be explained by the structure of the surface. No significant differences were observed between the different swabbing devices. Therefore according these results, the swabbing devices are suitable to be used as sampling device for detection of *L. monocytogenes* in food processing environments. However, detection can never be 100% guaranteed, therefore an appropriate sampling plan is necessary as well.

## References

[1] Blackman I.C., Frank J.F. (1996). Growth of *Listeria monocytogenes* as a biofilm on various food-processing surfaces. J. Food Protect..

[2] Cruz C.D., Fletcher G.C. (2011). Prevalence and biofilm-forming ability of *Listeria monocytogenes* in New Zealand mussel (*Perna canaliculus*) processing plants. Food Microbiol..

[3] Rodriguez A., Autio W.R., McLandsborough L.A. (2008). Effect of surface roughness and stainless steel finish on *Listeria monocytogenes* attachment and biofilm formation. J. Food Protect..

[4] Silva S., Teixeira P., Oliveira R., Azeredo J. (2008). Adhesion to and viability of *Listeria monocytogenes* on food contact surfaces. J. Food Protect..

[5] European Food Safety Authority (EFSA) (2006). The community summary report on trends and sources of zoonoses, zoonotic agents, antimicrobial resistance and foodborne outbreaks in the European Union in 2005. EFSA J..

[6] European Food Safety Authority (EFSA) (2007). The community summary report on trends and sources of zoonoses, zoonotic agents, antimicrobial resistance and foodborne outbreaks in the European Union in 2006. EFSA J..

[7] European Food Safety Authority (EFSA) (2009). The community summary report on trends and sources of zoonoses and zoonotic agents in the European Union in 2007. EFSA J..

[8] European Food Safety Authority (EFSA) (2010). The community summary report on trends and sources of zoonoses, zoonotic agents and food-borne outbreaks in the European Union in 2008. EFSA J..

[9] Gandhi M., Chikindas M.L. (2007). *Listeria*: A foodborne pathogen that knows how to survive. Int. J. Food Microbiol..

[10] Kells J., Gilmour A. (2004). Incidence of *Listeria monocytogenes* in two milk processing environments, and assessment of *Listeria monocytogenes* blood agar for isolation. Int. J. Food Microbiol..

[11] Winter C.H., Brockmann S.O., Sonnentag S.R., Schaupp T., Prager R., Hof H., Becker B., Stegmanns T., Roloff H.U., Vollrath G. (2009). Prolonged hospital and community-based listeriosis outbreak caused by ready-to-eat scalded sausages. J. Hosp. Infect..

[12] Gómez D., Ariño A., Carramiñana J.J., Rota C., Yangüela J. (2012). Comparison of sampling procedures for recovery of *Listeria monocytogenes* from stainless steel food contact surfaces. J. Food Protect..

[13] Carpentier B., Cerf O. (2011). Review—Persistence of *Listeria monocytogenes* in food industry equipment and premises. Int. J. Food Microbiol..

[14] Dimitrijevíc M., Anderson R.C., Karabasil N., Natasa P., Jovanovic S., Jelena N.T., Teodorovic V., Maja M., Dojcinovic S. (2011). Environmental prevalence and persistence of *Listeria monocytogenes* in cold-smoked trout processing plants. Acta Vet.-Beograd..

[15] Lianou A., Sofos J.N. (2007). A review of the incidence and transmission of *Listeria monocytogenes* in ready-to-eat products in retail and food service environments. J. Food Protect..

[16] Tompkin R.B. (2002). Control of *Listeria monocytogenes* in the food-processing environment. J. Food Protect.

[17] Keskinen L.A., Todd E.C.D., Ryser E.T. (2008). Impact of bacterial stress and biofilm-forming ability on transfer of surface-dried *Listeria monocytogenes* during slicing of delicatessen meats. Int. J. Food Microbiol..

[18] (2005). Commission Regulation (EC) No 2073/2005 of 15 November 2005 on Microbiological Criteria for Foodstuffs. Off. J. Eur. Communities..

[19] Gómez D., Ariño A., Carramiñana J.J., Rota C., Yangüela J. (2012). Sponge *versus* mini-roller for the surface microbiological control of *Listeria monocytogenes*, total aerobic mesophiles and *Enterobacteriaceae* in the meat industry. Food Control.

[20] (2004). Microbiology of Food and Animal Feeding Stuffs—Horizontal Methods for Sampling Techniques from Surfaces Using Contact Plates and Swabs.

[21] Tompkin R.B. (2004). Environmental sampling—A tool to verify the effectiveness of preventive hygiene measures. Mitt. Lebensm. Hyg..

[22] Nyachuba D.G., Donnelly C.W. (2007). Comparison of 3M^TM^ Petrifilm^TM^ Environmental *Listeria* plates against standard enrichment methods for the detection of *Listeria monocytogenes* of epidemiological significance from environmental surfaces. J. Food Sci..

[23] Schirmer B.C.T., Langsrud S., Moretro T., Hagtvedt T., Heir E. (2012). Performance of two commercial rapid methods for sampling and detection of *Listeria* in small-scale cheese producing and salmon processing environments. J. Microbiol. Meth..

[24] Vorst K.L., Todd E.C.T., Ryser E.T. (2004). Improved quantitative recovery of *Listeria monocytogenes* from stainless steel surfaces using a one-ply composite tissue. J. Food Protect..

[25] Carpentier B., Barre L. (2012). Guidelines on Sampling the Food Processing Area and Equipment for the Detection of *Listeria monocytogenes*. http://www.ansespro.fr/eurl-listeria/.

[26] Verran J., Redfern J., Smith L.A., Whitehead K.A. (2010). A critical evaluation of sampling methods used for assessing micro-organisms on surfaces. Food Bioprod. Process..

[27] Autio T., Hielm S., Miettinen M., Sjöberg A.M., Aarnisalo K., Björkroth J., Mattila-sandholm T., Korkeala H. (1999). Sources of *Listeria monocytogenes* contamination in a cold-smoked rainbow trout processing plant detected by pulse-field gel electrophoresis typing. Appl. Environ. Microbiol..

[28] Kovačević J., McIntyre L.F., Henderson S.B., Kosatsky T. (2012). Occurence and distribution of *Listeria* species in facilities producing ready-to-eat foods in Britisch Columbia, Canada. J. Food Protect..

[29] Food Safety and Inspection Service (FSIS) (2013). Isolation and Identification of *Listeria monocytogenes* from Red Meat, Poultry, Egg and Environmental Samples. http://www.fsis.usda.gov/wps/wcm/connect/1710bee8-76b9-4e6c-92fc-fdc290dbfa92/MLG-8.pdf?MOD=AJPERES.

[30] Moore G., Griffith C. (2007). Problems associated with traditional hygiene swabbing: The need for in-house standardization. J. Appl. Microbiol..

[31] Moore G., Griffith C. (2002). A comparison of surface sampling methods for detecting coliforms on food contact surfaces. Food Microbiol..

[32] Akier A., Denis R., Jacques G., Pierre M. (1990). Attachment of *Listeria monocytogenes* to stainless steel, glass, polypropylene, and rubber surfaces. J. Food Protect..

[33] Beresford M., Andrew P., Shama G. (2001). *Listeria monocytogenes* adheres to many materials found in food-processing environments. J. Appl. Microbiol..

[34] Kang D., Eifert J.D., Williams R.C. (2007). Evaluation of quantitative recovery methods for *Listeria monocytogenes* applied on stainless steel. J. Aoac Int..

[35] Takahashi H., Kuramoto S., Miya S., Kimura B. (2011). Dessication survival of *Listeria monocytogenes* and other potential foodborne pathogens on stainless steel surfaces is affected by different food soils. Food Control.

